# A review of the millipede genus *Sinocallipus* Zhang, 1993 (Diplopoda, Callipodida, Sinocallipodidae), with notes on gonopods monotony vs. peripheral diversity in millipedes

**DOI:** 10.3897/zookeys.90.1291

**Published:** 2011-04-14

**Authors:** Pavel Stoev, Henrik Enghoff

**Affiliations:** 1 National Museum of Natural History, 1, Tsar Osvoboditel Blvd, 1000 Sofia and Pensoft Publishers, 13a, Geo Milev Str., 1111 Sofia, Bulgaria; 2 Natural History Museum of Denmark (Zoological Museum), University of Copenhagen, Universitetsparken 15, DK-2100 København Ø, Denmark

**Keywords:** *Sinocallipus*, functional anatomy, gonopod monotony, troglomorphism, caves, southeast Asia, identification key, Pensoft Wiki Convertor

## Abstract

The millipede genus *Sinocallipus* is reviewed, with four new cave-dwelling species, *Sinocallipus catba*, *Sinocallipus deharvengi*, *Sinocallipus jaegeri* and *Sinocallipus steineri*, being described from caves in Laos and Vietnam. With the new records the number of species in the genus reaches six and the genus range is extended to Central Vietnam and North and Central Laos. Both, *Sinocallipus jaegeri* from Khammouan Province in Laos and *Sinocallipus simplipodicus* Zhang, 1993 from Yunnan, China, show high level of reduction of eyes, which has not been recorded in other Callipodida. Peripheral characters such as the relative lengths of antennomeres, the number of ocelli, the number of pleurotergites or even the shape of paraprocts and the coloration seem to provide more information for the distinction of the species than do the relatively uniform gonopods. The differences in gonopods mainly concern the shape and length of cannula, the length and shape of coxal processes *g* and *k*, and the number of the acicular projections of the femoroid. An explanation is offered for the function of the trochanteral lobe of 9th leg-pair. It provides mechanical support for the cannula and seems to assist sperm charge and insemination during copulation. An identification key to the species in the genus is produced to accommodate the new species. The new species descriptions were automatically exported at the time of publication to a wiki (www.species-id.net) through a specially designed software tool, the Pensoft Wiki Convertor (PWC), implemented here for the first time together with a newly proposed citation mechanism for simultaneous journal/wiki publications.

## Introduction

Callipodidans are still poorly documented in Southeast Asia, with only 15 species, four genera and three families being hitherto known in the region (Stoev et al. 2008). The family Paracortinidae Wang & Zhang, 1993 is richest in species and includes two genera, *Paracortina* Wang & Zhang, 1993 and *Angulifemur* Zhang, 1997, and 12 species ranging from the southern Chinese provinces Yunnan, Sichuan and Tibet in the North to the provinces Thanh Hoa and Hoa Binh in Vietnam to the South (Wang and Zhang 1993, Zhang 1997, Shear 2000, Stoev and Geoffroy 2004, Stoev et al. 2008). Being very obscurely diagnosed, *Angulifemur* will most likely be synonymised with *Paracortina* when types are re-examined and further materials become available for study (Stoev et al. 2008).

The family Caspiopetalidae Lohmander, 1931, which comprises eight species distributed mainly in Central Asia, south to Punjab in Pakistan, is known in Southeast Asia with only a single cave-dwelling species, *Bollmania beroni*Stoev & Enghoff, 2005, from Yan Dong Cave in Yunnan (Stoev and Enghoff 2005). The locality lies nearly 2500 km southeast from the nearest species, *Bollmania kohalana* (Attems, 1936) from Pakistan.

The third family, Sinocallipodidae Zhang, 1993, which is the only callipodid family entirely confined to the tropics, is considered to be the most primitive of all callipodidans and is placed in its own suborder, Sinocallipodidea (Shear 2000, Shear et al. 2003). Until now only two species of Sinocallipodidae have been described: *Sinocallipus simplipodicus* Zhang, 1993 from Xiao Cave in China, and *Sinocallipus thai* Stoev, Enghoff, Panha & Fuangarworn, 2007 from the surroundings of Sri Wilai Temple, Saraburi Province, Thailand (Zhang 1993, Stoev et al. 2007). Specimens provisionally assigned to *Sinocallipus simplipodicus* were recorded also from northern Vietnam (Enghoff et al. 2004) and southern Laos (Shear et al. 2003).

The biospeleological explorations of Dr Louis Deharveng and Mrs Anne Bedos (Muséum National d’Histoire Naturelle, Paris, hereafter MNHN) in caves in Vietnam, and the active collecting work of Dr Peter Jäger (Forschungsinstitut und Naturmuseum Senckenberg, Frankfurt, hereafter SMF) and Mr Helmut Steiner (Hanau, Germany) in Laos revealed new material of Callipodida which was kindly offered to us for study. All examined specimens turned out to belong to new species of *Sinocallipus*, which are described and illustrated below. In the paper we also comment on the gonopod shape in *Sinocallipus* and on some previously overlooked somatic characters, as well as the function of the trochanteral lobe on the 9th leg-pair – a structure lacking analogues in other callipodidans – in copulation. To facilitate the identification and better differentiation of the new taxa we also provide a key for their identification.

## Material and methods

All material treated in the paper is preserved in 70% ethanol and is shared between the MNHN, SMF and the National Museum of Natural History, Sofia (NMNHS). All photographs were taken with a Leica DFC 420 digital camera mounted on a Leica MZ16A stereomicroscope. Automontage Pro software from Syncroscopy was used for image-stacking 3D focus expansion. Drawings were made with the aid of a camera lucida mounted on Leica-WILD M10 and Leica-MZ16 microscopes. All illustrations were processed and additionally cleaned up with Adobe Photoshop CS.

All species descriptions are automatically exported at the time of publication to a wiki platform (www.species-id.net) through a specially designed software tool, the Pensoft Wiki Convertor (PWC), implemented here for the first time and described in this issue of ZooKeys (Penev et al. 2011). The link to each taxon’s wiki page is published in the paper, and vice versa, the citation of the original description is present on the top of the wiki page. The wiki environment allows a constant update of new information on the particular taxon. The citation of the wiki page includes always the original description of the taxon, along with the version number, date of creation and list of the contributors to the versioned wiki page.

## Taxonomy

### Genus Sinocallipus

Zhang, 1993

urn:lsid:zoobank.org:act:F13FC586-B6EE-47D2-AC23-A8C3AD66D3C7

http://species-id.net/wiki/Sinocallipus

Sinocallipus  Zhang, 1993, Proc. XI Int. Congr. Speleol. Beijing, 1993: 129. Type species: *Sinocallipus simplipodicus* Zhang, 1993, by original designation.

#### Emended diagnosis

(based on Shear et al. 2003): A genus of moderate-size Callipodida (40–70 mm); 55–72 pleurotergites (PT); with low, narrow, primary crests; secondary and tertiary crests absent; no crest transition or setal migration; setae thin and pointed, all in an anterior position. Leg-pairs 3–11 with coxal sacs. Head of males convex, pilose, without particular modifications. Organ of Tömösváry small, inconspicuous. Hypoproct tripartite, median sclerite largest, subtrapezoidal, bearing a pair of macrosetae; lateral sclerites each with a seta emerging from the posterior margin. First and second leg-pairs visibly shorter, third leg-pair only slightly shorter than succeeding legs; tarsi undivided; with a ventral comb-like series of setae. Tarsi divided from leg-pair 4 onwards. Second leg-pair in females unmodified. Vasa deferentia opening through gonopores on small protuberances on posterior side of the second coxae. Ninth legs in males with distomedial, deeply excavated trochanteral lobe bearing pointed projections. Gonosternum extending for the entire breadth of gonopods, lying basal to gonocoxae. Gonocoxa with two medial, clavate processes (*g* and *k*) and long, slender cannula (*ca*), cannula curved or coiled; femoroid (telopodite) without prostatic groove, with 2–4 slender, narrowly separated, terminal projections directed anteromediad and overlapping or terminating close to coxal processes.

### Sinocallipus catba sp. n.

urn:lsid:zoobank.org:act:0A26C350-DEF0-4FBE-AD31-2CD8E3681267

http://species-id.net/wiki/Sinocallipus_catba

Figs 1–5 26 30 

#### Material examined.

Holotype: ♂, 69 PT + telson, Vietnam, Hai Phong Province, Cat Ba Island, Hoa Cuong Cave near Gia Luang, 20.845161°N, 106.981597°E, 5 m alt., 30.IX.1998, by hand, L. Deharveng leg. VIET-485 (MNHN). – Paratype: ♂, 67 PT + telson, same island, Tien Duc Cave, 26.IX.1998, by hand, L. Deharveng leg., VIET-452 (MNHN).

#### Description of locality.

This species was found in two moderately long, humid caves. Tien Duc is approximately 100 m long, while Hoa Cuong is 100–120 m long. In Tien Duc, the specimen was found on non-humid walls. Both caves host a rich cave fauna, including unidentified cambalopsid and haplodesmid millipedes (L. Deharveng, in lit.).

#### Origin of name.

For Cat Ba Island, the type locality.

#### Diagnosis.

Differs from *Sinocallipus simplipodicus*, *Sinocallipus jaegeri* and *Sinocallipus thai* by the white-yellowish body colour, brown antennomeres 2–5 and eye composed of 33 ocelli, and from *Sinocallipus deharvengi* by the smaller body size, antennae and gonocoxal process *g*, as well as by having paraprocts divided into larger ventral and smaller dorsal sclerites.

#### Description.

##### Males:

**Figures 1–5.**
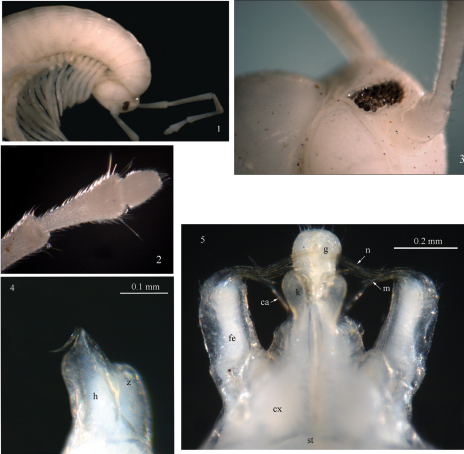
*Sinocallipus catba* sp. n.: **1** head and anteriormost pleurotergites **2** tip of antenna **3** ocelli **4** trochanter of leg-pair 9 **5** gonopods, anterior view. Abbreviations: cannula (ca); coxa (cx); coxal processes g and k; femoroid (fe); femoroidal acicular process (n); femoroidal subfalcate process (m); sternum (st); trochanter of leg 9: processes h and z.

Maximal length *ca* 50 mm, width of midbody PT 2.4 mm, 67–69 PT + telson. Body colour: uniformly white-yellowish, without particular coloration pattern; metazonites without posterior band. Head: uniformly white, cephalic suture visible. Antennae: long, extending beyond the posterior edge of PT 9 when folded backwards; antennomeres 2–5 light brown; 1, 6, 7 – white (Fig. 1); length of antennomeres: 1 – 0.3 mm, 2 – 1.4 mm, 3 – 1.8 mm, 4 – 1.2 mm, 5 – 1.3 mm, 6 – 0.7 mm, 7 – 0.4 mm; antennomere ratio: 3>2>5>4>6>7>1; tip of antennomere 7 with four cones protruding beyond posterior margin (Fig. 2). Eyes: black, well delineated, composed of 33 ocelli in 5 rows (Fig. 3).

Width of PT 2=3<1=4<5<6<7. PT higher than broad, ratio: 1.05 : 1. Dorsal side of collum and PT2–3 smooth, lateral sides ribbed. Crests poorly developed, broad and flattened anteriorly, abruptly narrowing and more pronounced posteriorly; 5+5 between the ozopores on midbody PT. Ozopores small, barely visible on most PT, lying between crests 5 and 6 in midbody PT. Paraprocts divided into smaller dorsal and larger ventral sclerites. Dorsal sclerite surmounted with two macrosetae in a vertical row. Spinnerets: long and slender, ending with a long seta.

All legs white-yellowish, long and slender, ending with a long claw. Tarsal pads very poorly developed, present on leg-pairs 3–12. No particular modifications on coxae of pregonopodal legs, prefemora of legs 4–7 swollen. Leg-pair 9 (Figs 4, 26): coxa subtrapezoidal; trochanter expanded medio-ventrad forming a rather elongated process (*h*) with a pointed tip and a smaller process *z*.

Chaetotaxy: unknown, all setae broken off.

Gonopods (Fig. 5): similar to those of congeners; coxae (*cx*): process *g* moderately large and swollen, *ca* 1.5 times the length of process *k*; processes *g* and *k* apically rounded, not truncated as in *Sinocallipus jaegeri*. Femoroid (*fe*): with three slender, acicular (*n*), and one short and subfalcate (*m*) terminal projections. Cannula (*ca*): long, and slender, not coiled.

##### Female:

unknown.

### Sinocallipus deharvengi sp. n.

urn:lsid:zoobank.org:act:376E58E2-1177-4A86-9F6D-ED22C99B36AA

http://species-id.net/wiki/Sinocallipus_deharvengi

Figs 6 12 27 30 

#### Material examined.

Holotype: ♂, 70 PT + telson, Vietnam, Quang Binh Province, Dong Hoi, Cha Noi: Hang Cha Noi (cave), 17.641363°N, 106.110375°E, 260 m alt., 8.I.1995, by hand, L. Deharveng & A. Bedos leg., VIET-064. – Paratypes: 1 juv., same locality, date and collectors; ad. ♀, 74 PT + telson, same province, Dong Hoi, Phong Nha: Hang Ruc (cave), 17.586134°N, 106.305667°E, 30 m alt., 6.I.1995, by hand, L. Deharveng & A. Bedos leg. VIET-059; 1 ♀, 70 PT + telson, same province, Dong Hoi, between Phong Nha and Cha Noi: Grotte de Troc, approx. coordinates: 17.6526°N, 106.243°E, about 70 m alt., 15.III.1997, L. Deharveng & A. Bedos leg. VIET-407 (all in MNHN).

#### Description of locality.

The species was found in several caves of moderate length (Hong Ruc about 50 m, Troc and Cha Noi more than 200 m), which seem to have never been surveyed previously. A road was built inside Hang Cha Noi during the War, and remains of rusted ammunitions were observed inside the cave at the time of collection. A rich cave fauna was found inside the Grotte de Troc, including two other species of Diplopoda, an undescribed cambalopsid, and *Eutrichodesmus asteroides* Golovatch et al., 2009 (Haplodesmidae) (Golovatch et al. 2009). All specimens were collected in the aphotic zone of the caves (L. Deharveng, in lit.).

#### Origin of name.

Named after the French zoologist Louis Deharveng, a passionate explorer of the caves of southeastern Asia, who together with A. Bedos collected this species.

#### Diagnosis.

Differs from congeners by having almost equally subdivided paraprocts, long antennae, strongly swollen and long gonocoxal process *g*, and comparatively short, pointed tip of the trochanteral process of leg 9. It can be distinguished from *Sinocallipus simplipodicus*, *Sinocallipus jaegeri* and *Sinocallipus thai* also by the white-yellowish body colour and brown antennomeres 2–5, and from *Sinocallipus catba* and *Sinocallipus steineri* by the large body size.

#### Description.

##### Males:

**Figures 6–9.**
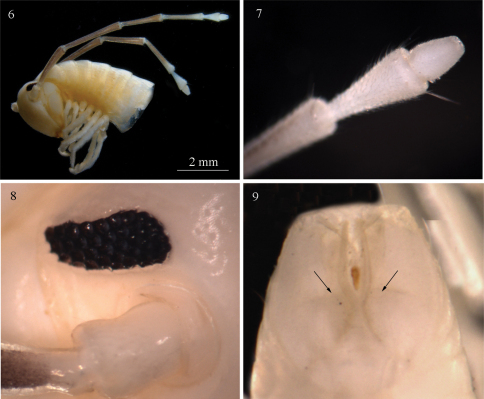
*Sinocallipus deharvengi* sp. n.: **6** head and anteriormost pleurotergites **7** tip of antenna **8** ocelli **9** telson, posterior view. Arrows on Fig. 9 show the division of paraprocts.

Maximal length: *ca* 68–70 mm, width of midbody PT 3.2 mm, 70 PT + telson. Body colour: uniformly white-yellowish, without particular coloration pattern, metazonites without posterior band. Head: uniformly white, pilose; cephalic suture visible. Antennae: long, extending beyond the posterior edge of PT 10 when folded backwards; antennomeres 2–5 light brown; 1, 6, 7 – white (Fig. 6); length of antennomeres: 1 – 0.5 mm, 2 – 2.0 mm, 3 – 2.5 mm, 4 – 1.7 mm, 5 – 1.8 mm, 6 – 0.8 mm, 7 – 0.4 mm; antennomere ratio: 3>2>5>4>6>1>7; tip of antennomere 7 with four short cones (Fig. 7). Eyes: black, well delineated, composed of 37–38 ocelli in 5–6 rows (Fig. 8).

Width of PT: 1=2=3<4<5<6<7. PT higher than broad, ratio: 1.06 : 1. Dorsal side of collum and PT 2–3 smooth. Crests poorly developed, flattened, 5+5 between the ozopores on midbody PT, anterior part of crests broad, abruptly narrowing posteriorly. Ozopores small, barely visible on PT 5–6, lying on crest 6 in midbody PT, missing on the last 4 PT. Paraprocts divided into two almost equal-sized dorsal and ventral sclerites (Fig. 9). Dorsal sclerite surmounted by a macroseta situated on a tiny lobe. Spinnerets: long and slender, ending with a long seta. All setae on telson dark brown, contrasting with the white background.

**Figures 10–12.**
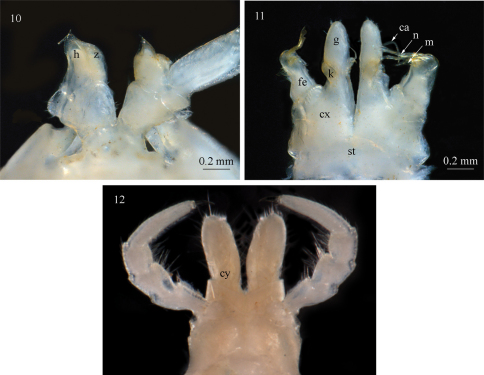
*Sinocallipus deharvengi* sp. n.: **10** trochanter leg-pair 9 **11** gonopods, anterior view **12** cyphopods and leg-pair 2. Abbreviations: cannula (ca); coxa (cx); coxal processes g and k cyphopods (cy); femoroid (fe); femoroidal acicular process (n); femoroidal subfalcate process (m); sternum (st); trochanter of leg 9: processes handz.

All legs white-yellowish, long and slender, ending with a long claw. Tarsal pads very poorly developed, present on leg-pairs 3–12. No particular modifications on coxae of pregonopodal legs. Prefemora of legs 4–7 swollen. Leg-pair 9 (Figs 10, 27): coxa subtrapezoidal; trochanteral lobe (*h*) with a comparatively short tip and poorly developed process *z*.

Chaetotaxy: unknown, all setae broken off.

Gonopods (Fig. 11): similar to those of congeners; differ by the large coxal (*cx*) process *g* more than 3 times the length of process *k*; processes *g* and *k* apically rounded, not truncated as in *Sinocallipus jaegeri*. Femoroid (*fe*): with three slender, acicular (*n*), and one short and subfalcate (*m*) terminal projections. Cannula (*ca*): long and slender, not coiled.

##### Females:

70–74 PT + telson; body colour darker, lateral sides light brownish; crests more pronounced than in males; second leg-pair unmodified (Fig. 12).

### Sinocallipus jaegeri sp. n.

urn:lsid:zoobank.org:act:BFC1EAC4-7CB3-4391-A203-2695AE2A596C

http://species-id.net/wiki/Sinocallipus_jaegeri

Figs 14–19 28 30 

#### Material examined.

Holotype: ad. ♂, 61 PT + telson; length *ca* 45 mm, width *ca* 1.90 mm; Laos, Khammouan Province, 9.5 km NE Thakek, 17°26.936N, 104°52.499E, 159 m alt., in foot cave, by hand, 31.X.2004, P. Jäger & V. Vedel leg. (SMF); – Paratypes: 2 ad. ♀♀ with 58 and 59 PT, same locality as holotype, 11.III.2007, P. Jäger & F. Steinmetz leg.; ♀, 59 PT, same locality, foot cave and surrounding, 28.X.2004, P. Jäger & V. Vedel leg.; juvenile, 42 PT, same locality and collectors, 30.X.2004, P. Jäger & V. Vedel leg.; ad. ♀ broken into pieces, more than 50 PT, Khammouan Province, Thakek area, Ban Tham, 17°25.799N, 104°51.906E, 161 m alt., jungle, trees, by hand, 31.X.2004, P. Jäger & V. Vedel leg. (all in SMF; one female in NMNHS).

#### Description of locality.

**Figure 13.**
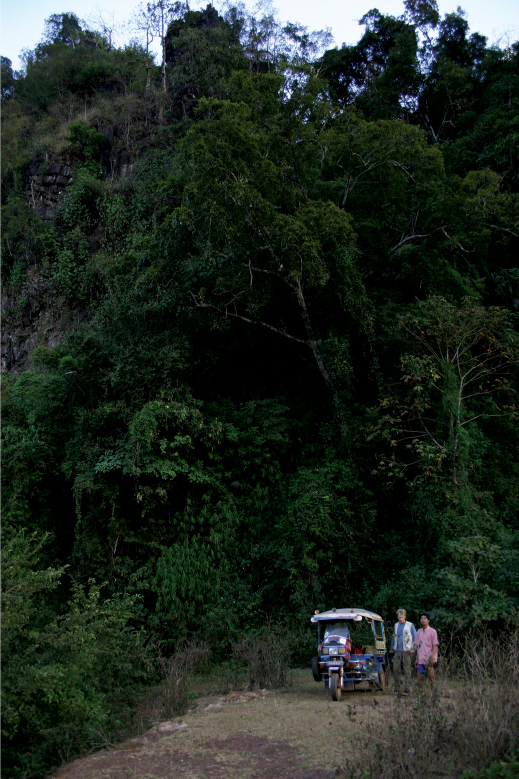
View of the type locality of *Sinocallipus jaegeri* sp. n. near Thakek, Laos (photo: P. Jäger).

Three of the adult specimens were found in a cave situated at the foot of a limestone hill overgrown with sparse vegetation (Fig. 13). They were collected in the aphotic zone of the cave, somewhere between 30 and 80 m from the entrance. The cave is at least 100 m long, wet, with dripping water and mud on the floor and partly on the cave walls. In the cave *Sinocallipus jaegeri* coexists with *Heteropoda maxima* Jäger, 2001 and *Sinopoda* sp. (Araneae: Sparassidae), *Thereuopoda longicornis* (Fabricius, 1793) (Chilopoda: Scutigeridae), and cave crickets (cf. Jäger 2007). One specimen was collected outside the cave, probably under stones close to the limestone hill, approx. 2 km SW of it where the other specimens were found.

#### Origin of name.

Named after Peter Jäger, curator of Arachnida and Myriapoda at SMF, who has been actively exploring the fauna of Laos since 2003 and collected the species.

#### Diagnosis.

Differs from congeners by the following set of characters: reduced eyes, composed of 10–11 ocelli; well expressed, narrow, pleurotergal crests; 59–61 PT; white-yellowish body and antennae; gonopods: process *g* short, almost half length of that of *Sinocallipus deharvengi*, apically truncated; *k* small and more abrupt apically than those of *Sinocallipus simplipodicus*, *Sinocallipus thai* and *Sinocallipus deharvengi*; cannula straight.

#### Description.

##### Males:

**Figures 14–19.**
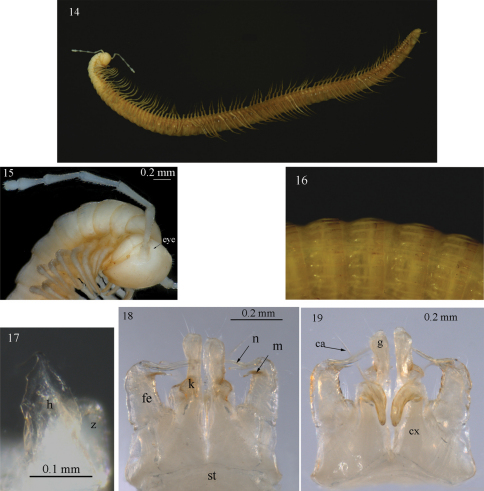
*Sinocallipus jaegeri* sp. n.: **14** habitus **15** close up of head and anteriormost pleurotergites **16** midbody pleurotergites, dorsal view **17** trochanter of leg-pair 9 **18–19** gonopods, anterior and posterior views, respectively. Abbreviations: cannula (ca); coxa (cx); coxal processes g and k; femoroid (fe); femoroidal acicular process (n); femoroidal subfalcate process (m); sternum (st); trochanter of leg 9: processes handz.

Maximal length *ca* 45 mm, width of midbody PT 1.7 mm, 61 PT + telson. Body colour: white-yellowish; head and anterior 10 PT much whiter than the gray-yellowish middle and posterior ones; metazonites with a posterior light brown band, more pronounced on posterior PT (Fig. 14). Head: uniformly white, pilose, cephalic suture barely visible. Antennae: long, slightly extending beyond the posterior edge of PT10 when folded backwards; length of antennomeres: 1 – 0.4 mm, 2 – 1.2 mm, 3 – 1.5 mm, 4 – 1.0 mm, 5 – 1.0 mm, 6 – 0.6 mm, 7 – 0.3 mm; antennomere ratio: 3>2>4=5>6>1>7; tip of antennomere 7 with four cones protruding well beyond the edge. All antennomeres snow white. Eyes: very inconspicuous, transparent, in adults composed of 10–11 small ocelli in two rows (Fig. 15).

Width of PT: 2–4<1=5<6<7<8<9<10. PT higher than broad, ratio: 1.06 : 1. Dorsal side of collum and PT 2–3 smooth; complete crests series appearing from PT 4 onwards. Crests at midbody PT well apart from each other; 6+6 (lateralmost one less pronounced), no secondary crest series, all crests well expressed (ribbed), narrow, equally broad along the whole length of metazonite, not touching anteriorly (Fig. 16). Ozopores situated on midbody PT between crests 3 and 4, visible from sixth to last but two PT. Paraprocts divided into smaller dorsal and bigger ventral sclerites. Each dorsal sclerite with a pair of macrosetae situated on small lobes in vertical line. Spinnerets: long and slender, ending with a long seta each. All setae on telson dark brown, contrasting with the white background.

All legs white-yellowish, very long and slender, ending with a long claw. Tarsal pads poorly developed, present only on leg-pairs 3–12. No particular modifications on coxae of pregonopodal legs. Prefemora of posterior legs less swollen than others. Leg-pairs 4–7: coxa subquadrate; prefemur strongly swollen. Leg-pair 9 (Figs 17, 28): coxa subquadrate; trochanter with two processes: anterior one (*h*) higher, leaf-shaped, its tip very sharp, spine-like, curved cephalad; posterior process (*z*) rounded with a small hook; a small pore (*p*) opening below the hook.

Chaetotaxy (Table 1): all setae slender and apically pointed, in anterior position on all segments (excluding penultimate ones).

**Table 1.** Chaetotaxy of anterior PT in *Sinocallipus jaegeri* sp. n.

	Anterior setae	Posterior setae
Collum	5+5	-
PT 2	5+5	-
PT 3	5+5	-
PT 4	5+5	-
PT 5	5+5	-
PT 6	5+5	-

Gonopods (Figs 18, 19): similar to those of congeners. Coxae (*cx*): process *g* short, almost half length of that of *Sinocallipus deharvengi*, apically truncated (in lateral view); *k* small and more abrupt apically than in *Sinocallipus simplipodicus*, *Sinocallipus thai* and *Sinocallipus deharvengi*. Femoroid (*fe*): with two slender, acicular (*n*), and one shorter and subfalcate (*m*) terminal projections, latter projecting into a long and thin filament. Cannula (*ca*): long and slender, not coiled.

##### Females:

58–59 PT in adults; middle PT slightly broader than those of the male.

### Sinocallipus simplipodicus

Zhang, 1993

urn:lsid:zoobank.org:act:817F9976-72F2-41A1-96C7-7A50E1A27FD4

http://species-id.net/wiki/Sinocallipus_simplipodicus

Fig. 30

Sinocallipus simplipodicus  Zhang, 1993, Proc. XI Int. Congr. Speleol. Beijing, 1993: 129, figs 1–16.
Sinocallipus simplicipodus  [sic!]: Wang and Mauriès 1996: 86. Shear 2000: 99.
Sinocallipus simplipodicus : Stoev et al. 2008: 7.

#### Distribution.

Only known from Xiao Cave, Hekou Yaozu Autonomous County, Yunnan Province, China.

#### Remarks.

Although callipodidans are often found in caves, especially in Southeast Asia and southern Europe, there are no species among them possessing an eye reduction similar to that observed in *Sinocallipus jaegeri* and *Sinocallipus simplipodicus*. Other peripheral characters, such as the elongated antennae and legs, in addition to the apparent depigmentation, also indicate their adaptation to the underground environment. A specimen of *Sinocallipus jaegeri* was found also outside caves, perhaps in deeper soil layers. The type specimens of *Sinocallipus simplipodicus* which are perhaps preserved in the Institute of Zoology, Chinese Academy of Sciences, or new topotypic material need to be examined to supplement the original description of Zhang which suffers from the poor quality of its illustrations.

### Sinocallipus steineri sp. n.

urn:lsid:zoobank.org:act:32886D5D-1D66-474E-B3E4-A27C7B845845

http://species-id.net/wiki/Sinocallipus_steineri

Figs 20–23 29 30

Sinocallipus : Steiner 2005, p. 96.

#### Material examined.

Holotype: ♂; 71 PT + telson, Laos, Luang Phrabang Province, Ponsai District, Ben Nambo (Thapo) Village, Tham Gia (Bat cave) (E-48-001/07), 19°57.233N, 102°25.457E, alt. approx. 400 m, 27.XII.2003, H. Steiner leg. (SMF); – Paratype: adult ♂, 67 PT, same locality, date and collector (NMNHS).

#### Description of locality.

For detailed descriptions of the cave and its exact locality see Burgers et al. (2005). The new species has been collected from the ceiling of the cave (H. Steiner, in lit.). Cave crickets, a spider, and the centipede *Thereuopoda longicornis* co-occur with *Sinocallipus steineri* (Steiner 2005, Jäger and Praxaysombath 2009).

#### Origin of name.

Named after the German biospeleologist Helmut Steiner, an active explorer of the caves of Laos, who collected the species.

#### Diagnosis.

Males: Differs from congeners by the following set of characters: 67–71 PT in adults; head and PT 1–4 white, remaining PT mottled light brown–grayish, antennomeres 2–6 brown; antennae moderately long, extending beyond posterior edge of PT7 when folded backwards; eyes black, well delineated, composed of 33–35 ocelli; midbody PT with 3+3 crests between ozopores; all crests flattened, almost equally broad along metazonal length, only slightly narrowed posteriorly and touching each other anteriorly. Gonopods: differ from those of congeners by the laterally narrowed gonocoxal process *g* and by the much longer process *k* being 2/3rd the height of *g*.

#### Description.

##### Males:

**Figures 20–23.**
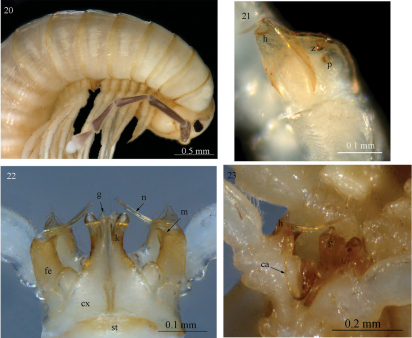
*Sinocallipus steineri*sp. n.: **20** head and anteriormost pleurotergites **21** trochanter of leg-pair 9 **22** gonopods, anterior view **23** gonopods and leg-pair 9 *in situ*, ventrocaudal view. Abbreviations: cannula (ca); coxa (cx); coxal processes g and k; femoroid (fe); femoroidal acicular process (n); femoroidal subfalcate process (m); trochanteral pore (p); sternum (st); trochanter of leg 9: processes handz.

Maximal length: *ca* 54–55 mm, width of midbody PT 2.5 mm, 67–71 PT + telson. Body colour: generally white-yellowish; head and PT 1–4 white, remaining PT mottled light brown–grayish, the last 1/5 of the body brownish; metazonites with a narrow transverse posterior band. Head: white-yellowish, pilose; epicranium marbled light brown; cephalic suture visible. Antennae: moderately long, extending beyond the posterior edge of PT 7 when folded backwards; antennomeres 1 and 7 white, 2–6 – brown; length of antennomeres: 1 – 0.4 mm, 2 – 1.1 mm, 3 – 1.3 mm, 4 – 0.8 mm, 5 – 0.9 mm, 6 – 0.7 mm, 7 – 0.3 mm; antennomere ratio: 3>2>5>4>6>1>7; tip of antennomere 7 with four cones protruding well beyond the edge. Eyes: black, well delineated, composed of 33–35 ocelli in 5 rows (Fig. 20).

Width of PT: 2–3<1=4<5<6<7<8<9<10. PT slightly higher than broad; ratio: 1.07 : 1. Dorsal side of collum and PT 2–3 smooth, ribbed only laterally; complete crests series appearing from PT 4 onwards. Midbody PT with 3+3 crests between ozopores; no secondary crest series, all crests flattened, almost equally broad along the metazonal length, only slightly narrowed posteriorly and touching each other anteriorly. Ozopores on midbody PT lying between crests 3 and 4, visible from sixth to last but two PT. Paraprocts divided into smaller dorsal and bigger ventral sclerites. Each dorsal sclerite with a pair of macrosetae in a vertical line. Spinnerets: long and slender, ending with a long seta each. All setae on telson dark brown, contrasting with the whitish background.

All legs white-yellowish, moderately long and slender, ending with a long claw. Tarsal pads poorly developed, present only on leg-pairs 3–12. No particular modifications on coxae of pregonopodal legs. Prefemora of legs 4–7 swollen. Leg-pair 9 (Figs 21, 29): coxa subtrapezoidal; trochanter with two processes: anterior one (*h*) higher, leaf-shaped, its tip very sharpened, spine-like, curved cephalad; posterior process (*z*) rounded with a small triangular bulge; a small pore opening (*p*) below the bulge.

Chaetotaxy: pleurotergal setae barely visible, minute, one each on PT 1 and 2, others presumably broken off.

Gonopods (Figs 22, 23): similar to those of congeners, but process *k* is longer. Coxae (*cx*): process *g* laterally narrowed, not clavate as in the other congeners; apical part slighly truncated (in lateral view); *k* 2/3 the height of process g, slightly bent lateralwards at midlength; apex with a small hook pointing towards process *g*. Femoroid (*fe*): with three slender, acicular (*n*), and one shorter and subfalcate (*m*) terminal projections, latter almost half length of the longest projection. Cannula (*ca*): long and slender, not coiled, its distal part lying between processes *h* and *z* on trochanter of leg 9 in close proximity to the pore opening (*p*).

##### Female:

unknown.

### Sinocallipus thai

Stoev, Enghoff, Panha & Fuangarworn, 2007

urn:lsid:zoobank.org:act:796391FA-D7CC-4701-9BB9-F4BFBF0C396A

http://species-id.net/wiki/Sinocallipus_thai

Figs 24 30

Sinocallipus thai  Stoev, Enghoff, Panha & Fuangarworn, 2007, Zootaxa 1450: p. 64, figs 1–7.
Sinocallipus thai : Stoev et al. 2008: p. 7.

#### Distribution.

Only known from the type locality in Thailand, Saraburi Province, Muang District, Sriwilai Cave Temple, 14°41'40"N, 100°54'34"E. The unique holotype was collected under a rock at the base of a limestone hill.

#### Remarks.

**Figure 24.**
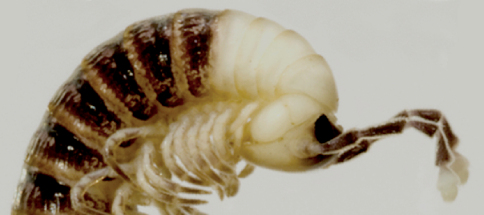
*Sinocallipus thai*:head and anteriormost pleurotergites (photo: G. Brovad).

This species is easily distinguished from congeners by its strikingly snow-white anterior pleurotergites and antennal tips, contrasting with a generally dark brown body (Fig. 24). The record of *Sinocallipus* cf. *simplipodicus* from southern Laos (Shear et al. 2003) may refer to this or a morphologically similar species (Stoev et al. 2008).

### Sinocallipus incertae sedis

Fig. 30

Sinocallipus  cf. *simplipodicus*: Enghoff et al. 2004: 36. Vietnam, Hanoi City, 1905, leg. Dr. Boutan, coll. A. Kempf.
Sinocallipus simplipodicus : Shear et al. 2003: 9, figs 3–14. Laos, Champasak/Attapu Provinces, Dong Hua Sao National Biodiversity Conservation Area, along the Houry Phak River near the SW edge of Bolavens Plateau, 15°04'37"N, 106°10'45"E, September 1999, H. Heatwole leg.

#### Material examined.

ad. ♀, Vietnam, Lang Son Province, Huu Lung Area, Snake cave, 19.III.1989, P. Beron leg. (NMNHS); 1 juv., same province and area, cave at 97 km N from Hanoi, 20.III.1989, P. Beron leg. (NMNHS).

## Discussion

*Sinocallipus* is remarkable in many aspects. In addition to its apparent primitiveness compared with other callipodidans, we here focus on the lack of noticeable gonopodal variation between species and the role of the 9th male legs during copulation.

### Gonopodal monotony vs. peripheral diversity

The genus *Sinocallipus* (as well as the monotypic family and suborder) can be defined by having each gonopod divided into a mesally expanded coxa bearing a cannula, and a telopodite (femoroid) without a prostatic groove bearing 2–4 acicular processes. The gonosternum lies at the base of the gonocoxae as in normal walking legs. In contrast to all other callipodidans which show considerable intrageneric variability in gonopod shape, gonopods in *Sinocallipus* are quite uniform. The differences mainly concern the shape and length of cannula, the length and shape of coxal processes *g* and *k*, and the number (2–4) of acicular projections of the femoroid.

**Figure 25.**
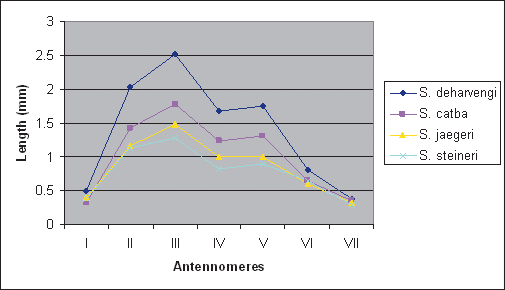
Graph showing antennomere lengths in the new species.

**Figures 26–29.**
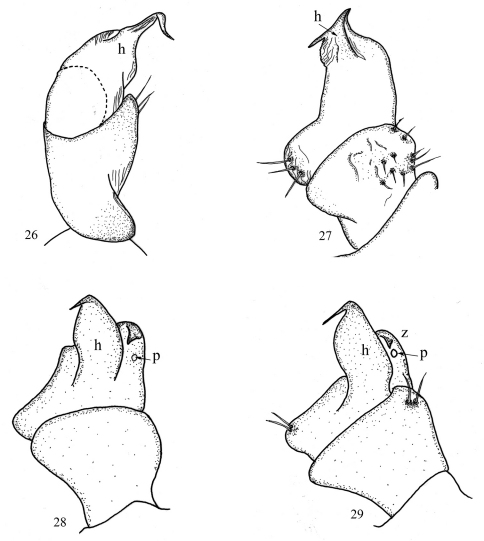
9th leg in *Sinocallipus*: **26** *Sinocallipus catba* sp. n. **27** *Sinocallipus deharvengi* sp. n. **28** *Sinocallipus jaegeri* sp. n. **29** *Sinocallipus steineri* sp. n. Abbreviations: process h; process z; pore (p).

In contrast, peripheral characters such as the relative lengths of antennomeres (Fig. 25), the number of ocelli, the number of pleurotergites or even the shape of paraprocts and the coloration seems to provide more information for the distinction of the species. There is a high degree of color variation in sinocallipods – from pale or light yellow-brownish in most of the species to dark brown in *Sinocallipus thai* (the only known specimen of which was collected outside a cave). Body size also varies substantially, from around 40 mm in *Sinocallipus simplipodicus* to more than 70 mm in *Sinocallipus deharvengi*. The same is true for the length of antennae where *Sinocallipus deharvengi* and *Sinocallipus jaegeri* on one hand and *Sinocallipus thai* on the other demonstrate the extreme cases (Table 2, Fig. 25). The shape of the trochanteral process of the 9th male leg-pair also varies and could be used as a species-specific character (Figs 26–29).

**Table 2.** Relative length of antennae in *Sinocallipus* species

Species	Antennae reaching back
*Sinocallipus catba*	beyond posterior edge of PT9
*Sinocallipus deharvengi*	beyond posterior edge of PT10
*Sinocallipus jaegeri*	beyond posterior edge of PT10
*Sinocallipus simplipodicus*	beyond posterior edge of PT8*
*Sinocallipus steineri*	beyond posterior edge of PT7
*Sinocallipus thai*	slightly beyond posterior edge of PT4

* inferred from fig. 1 in Zhang (1993).

*Sinocallipus* thus enters the ranks of millipede groups where the gonopods – often regarded as a *sine qua non* for species distinction in millipedes – are of little use for taxonomists while non-gonopodal (”peripheral”, ”somatic”) structures provide characters for identification. Hoffman (1981) called attention to what he called ”diphasic evolution” in polydesmidan millipedes: the difference between related species lies either in the exterior morphology, with gonopods showing little diversity, or in the gonopod structure, with peripheral characters showing less variation, see also Hoffman (1990). Examples of millipede groups with monotonous gonopods and diverse peripheral characters include the oxydesmid genus *Coromus* (Hoffman 1990), parts of the julid genera *Nepalmatoiulus*, *Dolichoiulus* and *Pachyiulus* (Enghoff 1987, 1992, Frederiksen, Petersen and Enghoff, unpubl.), the rhinocricid genus *Anadenobolus* (Bond and Sierwald 2002) and the harpagophorid genus *Thyropygus* (Pimvichai, Enghoff and Panha, unpubl.).

### Commentary on functional anatomy of gonopods and ninth male legs in *Sinocallipus*

Shear et al. (2003) stated that the cannula must represent the functional element in *Sinocallipus* copulation. According to these authors the most plausible method of spermatophore or seminal fluid transfer seems to be directly by the cannula which, being the longest gonopodal structure, could most easily contact the openings of the vasa deferentia to be “charged,” and subsequently penetrate the female cyphopods during copulation. Shear et al. (2003) found further support for this notion in the structure of telopodite which lacks a prostatic groove. However, no explanation has been offered for the function of the trochanteral lobe of 9th leg-pair – a structure lacking analogues in other callipodidans.

A close-up photograph of the gonopodal region of *Sinocallipus steineri* (Fig. 23) shows the distal part of the cannula embedded in the groove formed by the trochanteral processes on leg 9 in close proximity to the pore (*p*). This suggests that the 9th legs play a role in the copulation process, provided that the interpretation of Shear et al.’ is correct and indeed the cannula is the main structure used for sperm transfer. Besides mechanical support the trochanter probably provides secretions through the pore (*p*). However, until proven by direct observation, this statement remains speculative.

### Other unusual morphological traits

*Sinocallipus* exhibits further traits which were previously unknown in Callipodida. The division of each paraproct into a smaller dorsal and a larger ventral sclerite seems in general to show no variation within the order. However, in *Sinocallipus deharvengi* the paraprocts are divided exactly in the middle forming two nearly equal-sized halves (Fig. 9), while in all congeners they have the usual callipodidan shape. A character that might prove to be an autapomorphy for the genus is the presence of more than one seta (usually a pair, arranged in a vertical row) on the dorsal sclerite of each paraproct. This has been observed so far in four of the species and also in the female specimen of uncertain identity from the Snake cave, while it has not been recorded for the other species (where it might have been overlooked).

### Distribution

**Figure 30.**
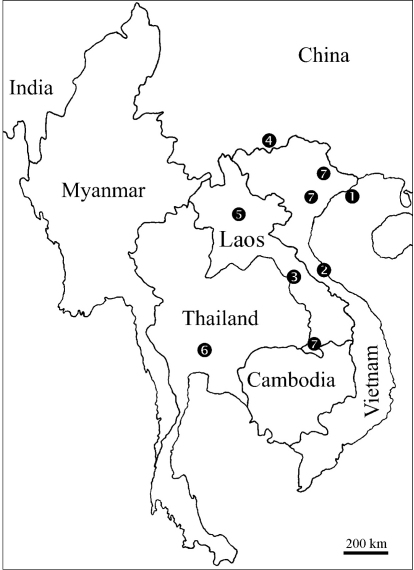
Distribution of the species of genus *Sinocallipus*: **1** *Sinocallipus catba* sp. n. **2** *Sinocallipus deharvengi* sp. n. **3** *Sinocallipus jaegeri* sp. n. **4** *Sinocallipus simplipodicus* **5** *Sinocallipus steineri* sp. n. **6** *Sinocallipus thai* **7** *Sinocallipus* spp.

*Sinocallipus* is the only genus in Callipodida entirely confined to the tropics, being hitherto known only south of the Tropic of Cancer, where its species are primarily confined to limestone caves and their surroundings. At present the genus range comprises the extreme South of China in Yunnan; Lang Son, Ha Noi, Quang Binh andHai Phong provinces in North and Central Vietnam; Saraburi Province in South Central Thailand; and Khammouan, Luang Phrabang and Champasak/Attapu provinces in Laos (Fig. 30). Shelley and Golovatch (2011) in their *magnum opus* on millipede biogeography presented updated maps of the distribution of the order Callipodida. These authors regard Callipodida as exhibiting an exclusively Laurasian distribution pattern and the occurrence of callipodidans in China and SE Asia as resulting from “spread into these areas after accretion of the “proto-southeast Asia” Gondwana I terranes”. Although their explicitly not phylogeny-based narrative is not entirely clear on this point, it seems that they regard the occurrence of callipodidans in China and SE Asia as secondary, resulting from dispersal from Laurasia. This explanation would imply a cladistically subordinate position of the Chinese and SE Asian callipodidans (see, e.g. Enghoff 1993) which, in the case of Sinocallipodidae, is in conflict with the assumed basal position of this family. A phylogenetic analysis is obviously needed in order to better understand the evolution and biogeography of the order Callipodida.

### Identification key to the species of *Sinocallipus*

**Table:** 

1(4)	body and antennae uniformly yellow-whitish; eye unpigmented, with less than 20 ocelli	2
2(3)	55–56 PT; 16 ocelli; crests low, flattened; cannula of gonopods short and coiled; Xiao cave, China	*Sinocallipus simplipodicus*
3(2)	59–61 PT, 10–11 ocelli; crests well-developed, ribbed; cannula long and straight; caves in Khammouan Province, Laos	*Sinocallipus jaegeri* sp. n.
4(1)	either body or antennae with brown pigment; eye black, with more than 30 ocelli	5
5(6)	body brown with light middorsal band stretching from PT 5 to the body end; first four PT, anterior part of head and antennal articles 6 and 7 snow white; eye with 45–50 ocelli; antennae short, slightly extending beyond posterior edge of PT 4, femoroid with two slender acicular and one shorter and subfalcate terminal projections; surroundings of Sri Wilai Temple, Thailand	*Sinocallipus thai*
6(5)	body generally white-yellowish, sometimes mottled gray-brownish; antennal articles 2–5 brown; antennae long, extending beyond the posterior edge of PT 7 when folded backwards; eye with less than 40 ocelli; femoroid with three slender acicular and one shorter and subfalcate terminal projections	7
7(8)	length of antennae *ca* 5.5 mm; 3+3 crests between ozopores on midbody PT, gonocoxal process *k* 2/3 length of process *g*; cave in Luan Prabang Province, Laos	*Sinocallipus steineri* sp. n.
8(7)	length of antennae more than 7 mm; 5+5 crests between ozopores on midbody PT; gonocoxal process *k* half length of process *g* or smaller; caves in Vietnam	9
9(10)	body length *ca* 70 mm; gonocoxal process *g* long, more than 3 times the length of process *k*; paraprocts divided into two almost equal in size sclerites; caves in Quang Binh Province, Vietnam	*Sinocallipus deharvengi* sp. n.
10(9)	body length *ca* 50 mm; gonocoxal process *g* long, *ca* 1.5 times the length of process *k*; paraprocts divided into larger ventral and smaller dorsal sclerites; caves on Cat Ba Island, Vietnam	*Sinocallipus catba* sp. n.

## Supplementary Material

XML Treatment for Sinocallipus

XML Treatment for Sinocallipus catba

XML Treatment for Sinocallipus deharvengi

XML Treatment for Sinocallipus jaegeri

XML Treatment for Sinocallipus simplipodicus

XML Treatment for Sinocallipus steineri

XML Treatment for Sinocallipus thai

XML Treatment for Sinocallipus
